# A Novel Electrical Equipment Status Diagnosis Method Based on Super-Resolution Reconstruction and Logical Reasoning

**DOI:** 10.3390/s24134259

**Published:** 2024-06-30

**Authors:** Peng Ping, Qida Yao, Wei Guo, Changrong Liao

**Affiliations:** 1 College of Aerospace Engineering, Chongqing University, Chongqing 400044, China; pingpeng@ntu.edu.cn; 2 School of Transportation and Civil Engineering, Nantong University, Nantong 226019, China; 2333320019@stmail.ntu.edu.cn; 3 Zhejiang Sanchen Electrical Company Limited, Lishui 323900, China; gw@sanchen.cn

**Keywords:** electrical equipment status diagnosis, image super-resolution, temporal information fusion, logical reasoning

## Abstract

The accurate detection of electrical equipment states and faults is crucial for the reliable operation of such equipment and for maintaining the health of the overall power system. The state of power equipment can be effectively monitored through deep learning-based visual inspection methods, which provide essential information for diagnosing and predicting equipment failures. However, there are significant challenges: on the one hand, electrical equipment typically operates in complex environments, thus resulting in captured images that contain environmental noise, which significantly reduces the accuracy of state recognition based on visual perception. This, in turn, affects the comprehensiveness of the power system’s situational awareness. On the other hand, visual perception is limited to obtaining the appearance characteristics of the equipment. The lack of logical reasoning makes it difficult for purely visual analysis to conduct a deeper analysis and diagnosis of the complex equipment state. Therefore, to address these two issues, we first designed an image super-resolution reconstruction method based on the Generative Adversarial Network (GAN) to filter environmental noise. Then, the pixel information is analyzed using a deep learning-based method to obtain the spatial feature of the equipment. Finally, by constructing the logic diagram for electrical equipment clusters, we propose an interpretable fault diagnosis method that integrates the spatial features and temporal states of the electrical equipment. To verify the effectiveness of the proposed algorithm, extensive experiments are conducted on six datasets. The results demonstrate that the proposed method can achieve high accuracy in diagnosing electrical equipment faults.

## 1. Introduction

Electrical and power equipment are widely used throughout the entire process of power generation, transmission, and control [1]. Electrical equipment operates continuously for extended periods, often in harsh environments, thus making it susceptible to aging and failure. Rapid detection and diagnosis of the state of electrical equipment are crucial for ensuring its reliable operation and maintaining the health of the entire power system [2].

Due to the wide distribution and complex states of electrical equipment, relying solely on manual inspection methods is insufficient to meet the development needs of the power system. An intelligent inspection strategy can leverage machine vision to quickly obtain the thermal state or pixel-level information of electrical equipment [3] and then extract the operational state of the electrical equipment using deep learning-based methods [4,5]. However, several challenges exist in acquiring state information or fault characteristics of electrical equipment through machine vision. First, machine vision technology requires high-precision images to accurately recognize the fault characteristics of electrical equipment. However, the complex structure of electrical equipment, with its various irregular shapes and surface textures, increases the difficulty of image processing and feature extraction. Secondly, electrical equipment typically operates in challenging environments, such as high temperature, high pressure, and high humidity. These environmental factors can degrade image quality through blurring and noise, thus affecting the performance of the machine vision system [6]. Additionally, changes in lighting conditions can create further difficulties in image acquisition and processing [7]. All these factors present significant challenges to the stability and reliability of machine vision-based fault diagnosis algorithms.

However, most current image-based fault detection methods do not consider the clarity of the images, thus making it difficult to accurately identify subsequent equipment faults. In addition, deep learning-based fault diagnosis methods often lack effective logical reasoning capabilities and can only use abnormal patterns in images to capture machine faults. This results in relatively weak logical reasoning capabilities for diagnosing faults during actual operation. Some faults in electrical equipment require logical reasoning to detect, and it is challenging to identify such faults directly through visual image recognition, such as abnormal switch statuses and panel indication misalignments. How to coordinate the fusion detection of multiple anomalies is a problem that needs to be solved in current electrical equipment fault detection [8].

To address the above challenges, we first design a super-resolution reconstruction method based on GAN [9] to remove noise from images of electrical equipment and perform high-definition reconstruction on images with varying brightness, darkness, and degrees of blurring. Then, using a deep learning method, we analyze the pixel features of the electrical equipment to determine its actual operational status. Finally, we construct an interpretable fault logic reasoning method for power equipment clusters based on logic diagrams. Experimental validation shows that the method proposed in this paper can improve the fault recognition accuracy of electrical equipment and provide interpretability in fault diagnosis.

The contributions of this paper are as follows:

1. An image super-resolution reconstruction method based on the GAN is proposed, which introduces a third-order image degradation model to simulate the real image quality degradation process during image acquisition. A U-Net discriminator with spectral normalization is also adopted to enhance the network’s ability to process complex images. This method significantly improves the quality of power equipment images and provides robust data support for the detection of an abnormal status of power equipment.
2. A method for deriving system state transition based on C-AOG is proposed, which uses a multilayer Causal And-Or Graph Model (C-AOG) to represent the equipment state transition model under the collaborative operation of multiple equipment events, thus allowing for the inference of the specific operating status of the power system.
3. An interpretable fault logic reasoning method for power equipment clusters based on logic diagrams is proposed, which adopts the Backpropagate Through Logic (BPTL) algorithm to address incorrect or self-conflicting formulas that may arise during the inference of system state transitions based on the causal diagram architecture. This ensures that the inferred transitions consistently adhere to true rules.

The remainder of the paper is organized as follows. Section 2 provides a brief overview of related work in the area of pattern recognition and fault diagnosis for electrical equipment. The details of the proposed methods are outlined in Section 3, followed by the experiment setup and results presented in Section 4. Finally, Section 5 presents the conclusion and discussion of this study. Table 1 lists the acronyms used in the paper.

## 2. Related Work

### 2.1. Abnormal State Detection Method for Electrical Equipment

Current methods for the abnormal state detection of electrical equipment encompass two major categories: traditional methods and artificial intelligence-based methods. Traditional methods primarily rely on physical models and empirical rules [10], including threshold-based methods, statistics-based methods, and feature engineering-based methods. In contrast, artificial intelligence-based methods include machine learning and deep learning techniques. Threshold-based methods identify equipment faults by setting specific thresholds to determine whether the equipment parameters are out of normal range [11]. Although this method is simple, it struggles to handle complex faults and noise disturbances. Statistical-based methods, on the other hand, utilize statistical principles to analyze equipment parameters such as mean, variance, correlation, and other indicators to identify abnormalities [12]. However, this method imposes high data requirements and struggles with nonlinear relationships. Feature engineering-based methods extract the features of equipment parameters, such as spectral features and time domain features, and then employ machine learning algorithms for classification or regression to achieve fault detection [13]. This approach requires manual design of the features and often relies on the experience of domain experts. Machine learning-based approaches use machine learning algorithms, such as support vector machines (SVMs), decision trees, and random forests, to model and classify equipment parameters for fault detection [14]. While this approach can automatically learn patterns and regularities in the data, it is more sensitive to data quality and feature selection. Deep learning-based methods utilize deep neural network structures [15], such as convolutional neural networks (CNNs) [16], recurrent neural networks (RNNs) [17], and long-short-term memory networks (LSTMs) [18], to learn features directly from raw data and conduct fault identification. This method excels inhandling large-scale data and complex features, thus possessing robust representational and generalization capabilities.

Most of the aforementioned methods for detecting the abnormal states of equipment using images for electrical equipment fault detection algorithms are based on the assumption that electrical equipment images exhibit high-quality accuracy for fault detection [19]. However, in practice, electrical equipment images are frequently influenced by various external factors, such as weather conditions, changes in lighting, and shooting angles [20], which may lead to local noise or overall blurring of the image. This degradation in the image quality directly affects the accuracy and efficiency of fault recognition. Therefore, image resolution reconstruction needs to be considered to enhance the accuracy and robustness of fault recognition.

In addition, deep learning-based fault diagnosis methods typically rely on a large volume of image data for training, with their models primarily emphasizing the abnormal features within images. Consequently, these deep learning methods often struggle to logically derive fault states and conduct in-depth analysis and explanation of fault causes [21]. This leads to low interpretability of the fault diagnosis results, which may not be sufficiently effective for actual maintenance and repair work. Therefore, detecting multiple anomalous states through logical reasoning with synergistic fusion poses a challenging problem in the current field of electrical equipment fault detection. Introducing logical reasoning methods can enhance the understanding of the causes and mechanisms behind fault occurrences, thereby improving the accuracy and reliability of fault diagnosis.

### 2.2. Image Super-Resolution Reconstruction Methods

The present image super-resolution reconstruction methods are mainly categorized into traditional and deep learning-based methods. Traditional image super-resolution methods mainly include interpolation-based, reconstruction-based, and learning-based methods. Interpolation-based methods use interpolation techniques, such as one-dimensional nearest neighbor, linear, and cubic interpolation, which can improve image magnification but may lead to uneven image boundaries and mosaic effects [22]. Reconstruction-based super-resolution methods utilize unbalanced and balanced sampling theorems to improve the spatial resolution of the image by addressing the aliasing problem, e.g., frequency domain methods and spatial domain methods [23]. In contrast, learning-based methods use a large number of images to train a super-resolution reconstruction model. This allows the model to learn a priori knowledge and recover high-frequency details. These methods typically involve two main steps: training and reconstruction [24].

Traditional image super-resolution reconstruction methods, though diverse, perform poorly in complex situations. With the rise of deep learning, image super-resolution has seen a new development. Instead of tedious feature engineering, deep learning methods learn image features directly. Among them, methods based on convolutional neural networks have made significant breakthroughs. Dong et al. proposed SRCNN [25], but its structure is relatively simple. Subsequent improvements include FSRCNN [26], VDSR [27], and so on, which led to better reconstruction and fewer network parameters [28]. Meanwhile, utilizing the advantages of the Laplace pyramid network, LapSRN [29] achieved significant results in complex scenes. In addition, Ledig et al. proposed SRGAN, which utilizes generative adversarial networks to suppress the shortcomings of traditional techniques [30] and achieve more accurate reconstruction results.

### 2.3. An Interpretable Electrical Equipment Fault Logic Reasoning Method Based on Time Series Fusion

The advancement of intelligence and automation in power equipment, fault diagnosis, and prediction have emerged as crucial tasks in equipment maintenance and management. Traditional fault diagnosis methods typically rely on expert experience and sensor data analysis, thus leading to issues such as low accuracy, unreliability, and susceptibility to interference. Therefore, researchers have begun to focus on the logical derivation method of interpretable electrical equipment faults based on time series fusion. This method first collects time series data of power equipment, including parameters such as current, voltage, temperature, etc., and stores them in a database. Then, using data mining and machine learning techniques, these data are analyzed and processed to extract the operational status and fault characteristics of the equipment. Then, through time series fusion technology, data from different periods are integrated to form comprehensive information about the equipment’s working state. For instance, Chen et al. [31], proposed an innovative semantic fault diagnosis method based on temporal logic, thus aiming to address the increasing complexity and vast amount of time series data in Industrial Internet of Things (IIoT) systems. By constructing a formal specification of faults, the method not only improves the accuracy of fault diagnosis but also enhances the interpretability of the diagnostic results, thereby enabling humans to intuitively understand fault situations.

For event-based causation analysis, the Structural Causal Model (SCM) stands out as a more efficient causal inference method. The SCM integrates concepts from structural equations, virtual factual models, and probabilistic graphical models, thus applying them to causal analysis [32]. Recently, causal inference models based on Bayesian networks or syntactic-directed acyclic graphs have garnered increasing attention from researchers for analyzing complex interaction behaviors and inferring system state changes. Research findings indicate that causal graph models offer promising applications in reasoning about the evolution of observations in observation-constrained scenarios [33,34,35]. Typical causal inference models include Bayesian networks, temporal analysis networks, directed graph models, and other structures. In this paper, causal methods and graphs should be revised as causal inference methods to establish the correlation between the visual recognition results and the state of the complex system [36], thus aiming to develop an interpretable fault diagnosis model for power equipment.

The interpretable electrical equipment fault logic reasoning method based on time series fusion can enhance the accuracy and reliability of fault diagnosis, thus offering robust support for the maintenance and management of electrical equipment. In the future, as artificial intelligence and big data technology continue to evolve, this method is poised to see broader adoption and deeper exploration.

## 3. Methods

### 3.1. Quality Enhancement of Electrical Plant Images

#### 3.1.1. Light Balancing Methods for Images

In image processing, a common challenge is to balance the brightness of pictures with areas that are too dark or too bright. This can be achieved through dynamic brightness adjustment. Firstly, by analyzing the brightness histogram of the image, we can understand the distribution of brightness. Based on this distribution, we design a conversion function that can adjust the brightness of a pixel according to its current brightness value. For bright pixels (x>H), we can use a linear function with a slope less than 1 to reduce its brightness. f(x)=x−α∗(x−H), where *x* represents the original pixel’s brightness value, f(x) represents the adjusted brightness value, *H* is the high-brightness threshold, and α is the adjustment intensity. Similarly for dark pixels (x<L), we can use a linear function with a slope less than 1 to increase their brightness levels. f(x)=x+β∗(L−x), *L* is the dark brightness threshold, and β is the adjustment intensity. For pixels of intermediate brightness, the selection (L≤x≤H) is kept constant. Then, we apply the conversion function f(x) to each pixel p_(i,j) in the image, where i,j are the row and column indexes of the pixel, respectively. After adjusting the brightness of the image, we use a smoothing filter to reduce some of the noise caused by this operation, thereby making the image look clearer and smoother.

#### 3.1.2. Super-Resolution Reconstruction Methods for Images

**Figure 1:**
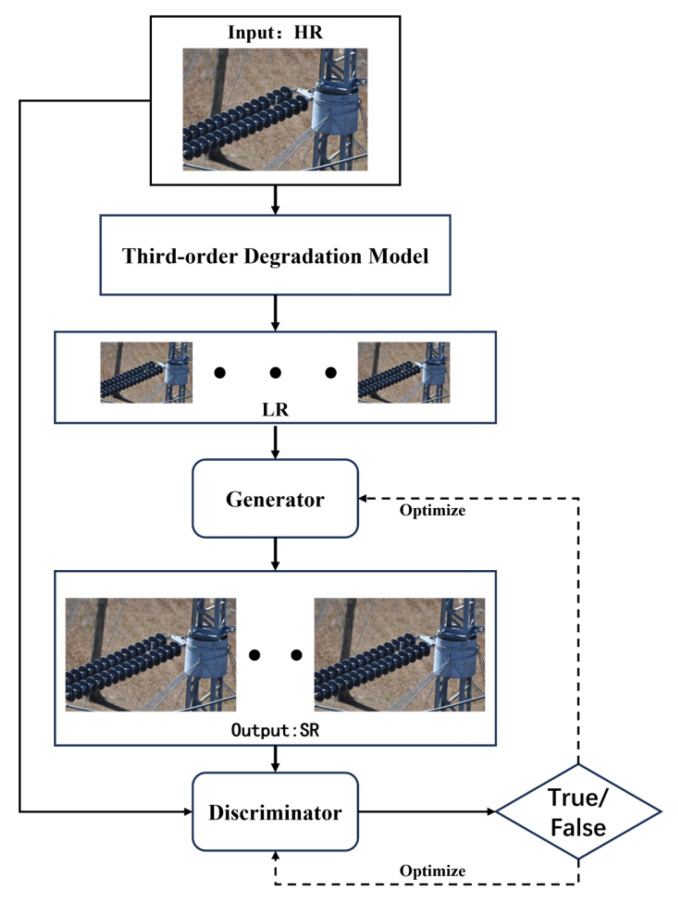
Flowchart of the super-resolution reconstruction method.

In practice applications, the acquisition of electrical equipment images is often affected by various factors, thus resulting in suboptimal image quality. In particular, due to weather changes, such as rain, snow, haze, and other unfavorable conditions, these may interfere with the image acquisition process, thereby resulting in blurring, color distortion, and other problems in the image. In addition, capture devices may experience jitter during operation for various reasons, thus resulting in noise in the image, which can significantly affect image quality and usability. Given the above problems, there is an urgent need for an effective image processing method to remove these noises and improve image quality. To address this need, we propose an improved super-resolution reconstruction method, whose overall flow is depicted in Figure 1. This method not only denoises the image but also generates clearer and more detailed high-resolution (HR) images through reconstruction techniques.

**Figure 2:**
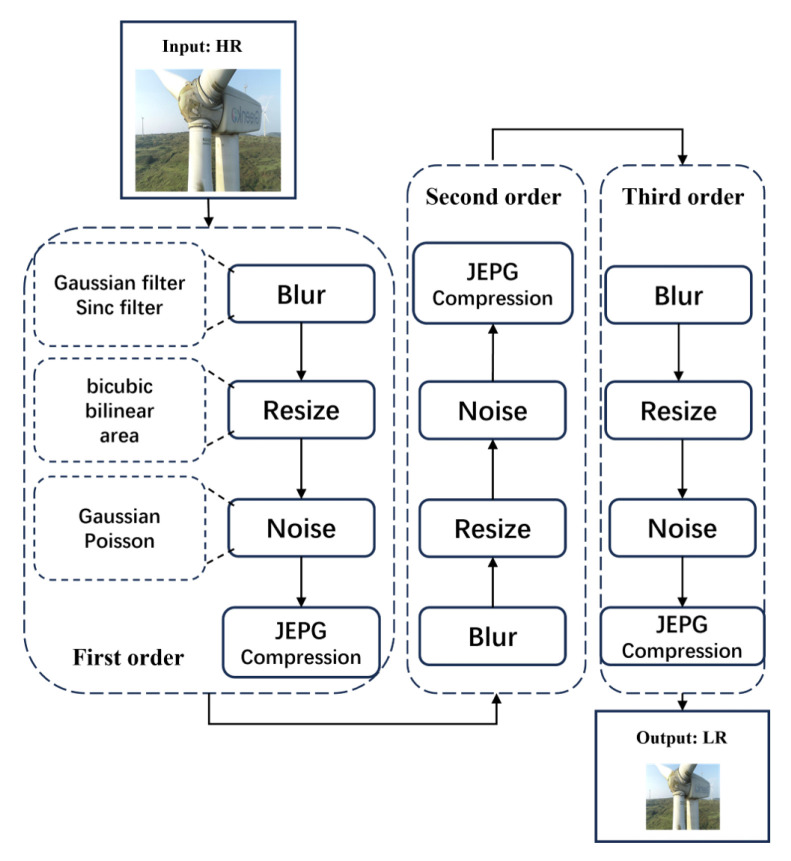
Third-order degradation model for electrical equipment images.

In comparison to previous super-resolution reconstruction methods, we have developed a novel approach by first constructing a third-order degradation model for electrical equipment images. This model simulates the real degradation process, thus producing low-resolution images that closely resemble actual degradation. The process flow is illustrated in Figure 2 and comprises four phases: blurring, stochastic scaling, noise addition, and JPEG compression. The blurring phase encompasses Gaussian and sinc blurring methods, while the stochastic scaling phase includes bicubic, bilinear, and area scaling techniques. Furthermore, the noise addition phase introduces Gaussian and Poisson noise types. Degradation kernels for these processes are randomly selected and applied in multiple iterations to mimic real-world conditions.

**Figure 3:**
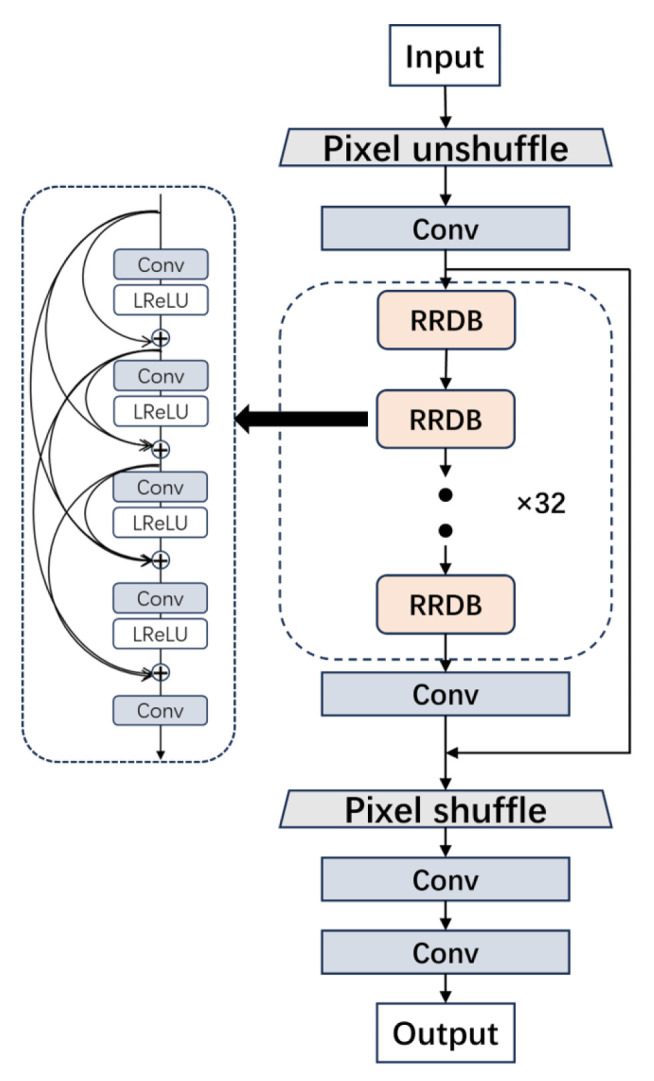
Generator network structure.

The generator of the super-resolution reconstruction network includes a two-pixel module, four convolutional layers, and a residual dense module group. The Residual-in-Residual Dense Block (RRDB), consisting of 32 residual-in-residual dense blocks, is used to obtain deeper and more complex structures. Each RRDB comprises five convolutional layers and four LeakyReLU activation function layers, with a convolution kernel size of 3 × 3. To better preserve details and enhance image clarity and quality, traditional upsampling and downsampling layers are replaced with Pixel shuffle and Pixel unshuffle methods. This modification improves the performance of the super-resolution reconstruction network. The generator structure is shown in Figure 3.

**Figure 4:**
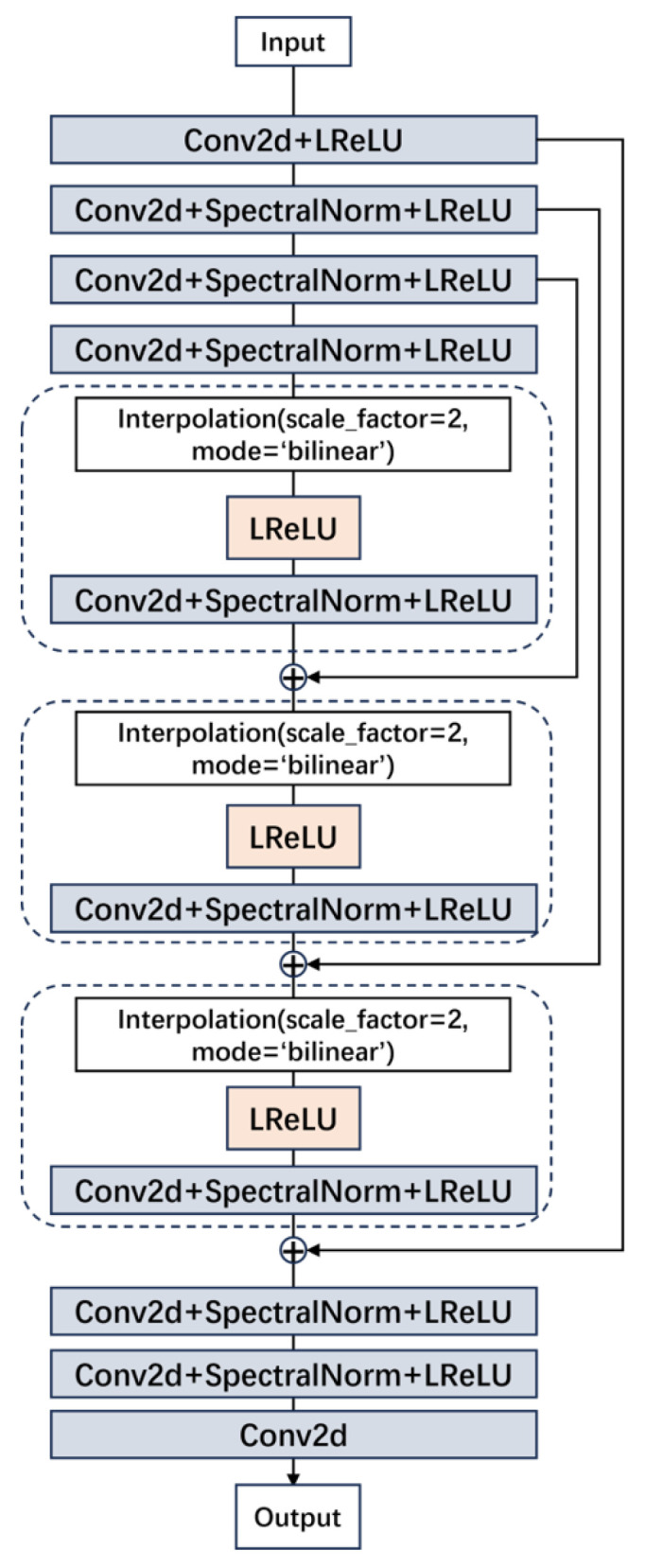
Discriminator network structure.

In the discriminator of the super-resolution reconstruction network, like Wang et al. [37], we adopted the U-Net discriminator containing spectral normalization, which includes a downsampling module, three upsampling modules, and an output module. The downsampling module includes a convolution layer and three layers of convolution with spectral normalization, and the upsampling module includes a bilinear interpolation, a LeakyReLU activation function layer, and a convolution layer with spectral normalization. The output module includes two layers of convolution with spectral normalization and a convolution output layer. By adopting this discriminator, the network’s ability to process complex image data is further enhanced, and the training stability and model generalization ability are also improved. The structure is shown in Figure 4.

By utilizing an improved super-resolution reconstruction method, we have effectively mitigated the adverse effects of factors such as weather conditions and device jitter on the image quality of the electrical equipment. This method yields a clearer and more accurate depiction of the actual equipment condition, thus offering reliable visual support for power system operation monitoring and equipment maintenance. Specifically, the improved super-resolution reconstruction technology enhances image quality at the pixel level, thereby making details more discernible. This aids in the timely detection of wear and tear, damage, or other potential issues on the equipment’s surface. The application of this technique not only boosts the efficiency of electrical equipment monitoring and maintenance but also provides more reliable and comprehensive data support for related research and applications. Ultimately, it promotes the safe and stable operation of power systems.

### 3.2. A Joint Learning-Based Interpretable Logic Reasoning Method for Power Equipment Faults

In the process of in-depth research on electrical equipment fault reasoning, we recognize that traditional reasoning methods are often inadequate when facing complex and changing fault patterns. In order to solve this problem more accurately and efficiently, we adopted a time series-based knowledge graph reasoning method. This method not only fully utilizes the advantages of knowledge graphs in representing and storing complex relationships, but it also introduces temporal information to make the inference process more closely related to the dynamic process of actual fault occurrence.

Specifically, the time series-based knowledge graph reasoning approach enables us to construct a comprehensive and systematic knowledge base of electrical equipment faults. This knowledge base represents various fault modes, causes, effects, and solutions in the form of a graph. Such a representation is not only intuitive and easy to understand, but it also clearly demonstrates the correlation between faults and the evolution process. Furthermore, by incorporating temporal information, we can precisely describe the sequence, duration, and trend of faults. This enables more accurate inference of fault causes and potential solutions.

In power equipment, system-level faults often arise from abnormal state transitions or clustered failures across multiple nodes. Consequently, this study investigates a fault state reasoning method based on logical reasoning to facilitate interpretable learning of power equipment faults. The state derivation structure consists of bottom-up derivation of system state transitions based on a causal graph architecture, and the state interpretability is realized through a top-down BPTL framework for fault causation prediction.

#### 3.2.1. A Method for Deriving System State Transition Based on C-AOG

Firstly, we encoded the atomic state g based on the device’s state expression and combine multiple atomic states to form event e. We use G and E to represent the atomic state and event set of a certain device, respectively. Specifically, the switch status of the equipment is expressed as 0–1, and the fault state of a device (such as damage or overheating) is characterized by detection probability and temperature distribution. The event set formed by atomic states allows us to directly detect device faults such as wrong switch position, device overheating, etc. However, how individual devices spread faults requires further formation of a high-dimensional logical inference network to proceed. In our study, we consider the state information of electrical devices in the same layer as a timestamped Static Knowledge Graph (SKG) sequence, $G=\{G_{1},G_{2},...,G_{t},...\}$. Each SKG in G is denoted as $G_{t}=\left\{N,R,T\right\}$. N,R,T denote the state set, relation set, and event set, respectively. $G_{t}$ consists of a set of events with the same timestamp *t*. Events in $G_{t}$ can be represented as timestamped quartets, i.e., (Subject, Relation, Object, Time), and the quartets (s,r,o,t)∈G, s∈E, r∈R, o∈E, t∈T. The event set *E*, on the other hand, is a collection of event occurrences under the knowledge graph data characterization, where *E* mainly contains the loads under the switching changes, as well as the load changes caused by faults. The probability of load change $p_{\mathrm{load}\;}$ for all device nodes can be calculated using the following Equation (1):

(1) $$p_{\mathrm{load}\;}=p\left(s|H_{t-1},R_{t-1},o,r\right)$$

where $H_{t-1}$ and $R_{t-1}$ denote the embedding representation matrices of entities and relations, respectively, at the moment t−1.

**Figure 5:**
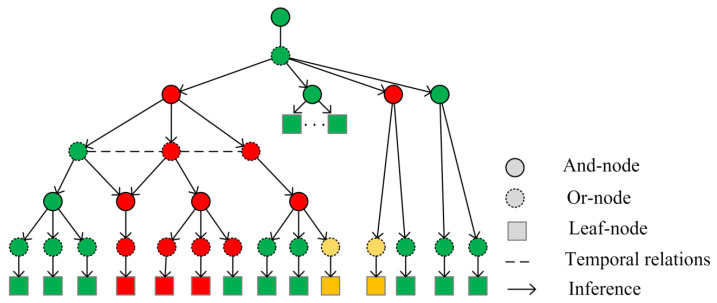
Logical hierarchical construction of equipment state change. Different colors in the boxes represent the different status of the electrical devices. And the colors in the and-or node represent the trigger state; red means not triggered and green means triggered.

We utilized the multilayer Causal And-Or Graph Model (C-AOG) to represent the equipment state transition model under the collaborative operation of multiple equipment events, thus facilitating the inference of the specific operating status of the power system. The specific logical structure extraction is based on general FOL to abstract the C-AOG graph, and we set the middle layer as and/or logical structure to realize the logical hierarchical construction of the graph. This is shown in Figure 5.

For the construction of specific graph logic levels, we use a combination of neural perception and logical reasoning for optimization.

Define the input as semantic data *x*, e.g., various electrical devices (switchgear, transformers, fans, transmission cables, etc.); Attributes *z*, e.g., attributes of the electrical devices (e.g., color, function, brightness of the switchgear buttons); and given a label y∈{0,1}, we denote whether or not the attribute *z* of the input *x* satisfies a certain latent logical concept.

The goal of neural symbolic learning is to simultaneously learn p(y∣x) and explicitly discover the correct logical concept $\Delta^{*}$, as well as the attribute mapping function from *x* to *z*. To enhance the performance and interpretability of neural logic inference, p(y∣x) is decoupled into two parts, which are neural perception and symbolic inference:

(2) $$p\left(y|x\right)=\sum_{z\in Z}p_{\theta }\left(z|x\right)\cdot p_{\phi }\left(y|\left\{z_{i}\right\}\right)$$

where the attribute mapping $p_{\theta }\left(z|x\right)$ is a neural network, and the logic module $p_{\phi }\left(y|\left\{z_{i}\right\}\right)$ is modeled using the symbol system $\Delta_{\phi }$. Here, *z* is rewritten as $z_{i}$ to emphasize the role of each attribute $z_{i}$.

(3) $$\begin{array}{cc} \; & p_{\theta }\left(z|x\right)=\prod_{i}\sigma {\left(nn_{\theta ,i}\left(x\right)\right)}_{z_{i}}\\ \; & =\prod_{i}\frac{\exp\left(nn_{\theta ,i}{\left(x\right)}_{z_{i}}\right)}{\sum_{z^{\prime }\in Z_{i}}\exp\left(nn_{\theta ,i}{\left(x\right)}_{z^{\prime }}\right)} \end{array}$$

where $nn_{\theta ,i}\left(x\right)$ is a DNN with $|Z_{i}|$ outputs.

(4) $$p_{\phi }\left(y|z\right)=\left\{\begin{array}{cc} 1, & \Delta_{\phi }\left[\left\{z_{i}\right\}\right]⊧y\\ 0, & \;\mathrm{otherwise}\; \end{array}\right.$$

where $\Delta_{\phi }\left[\left\{z_{i}\right\}\right]⊧y$ means that $\Delta_{\phi }\left[\left\{z_{i}\right\}\right]$ is consistent with y.

#### 3.2.2. Backpropagate through Logic-Based Fault Causation Inference Method for Electrical Equipment

**Figure 6:**
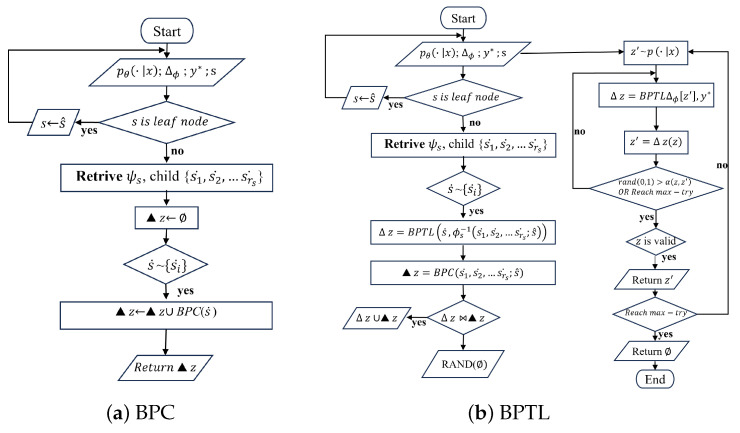
The workflow diagram of Algorithm.

Given that the inference of system state transitions based on causal diagram architecture may sometimes yield incorrect or even self-conflicting formulas, we employed the Backpropagate Through Logic (BPTL) algorithm [38] to solve the problem by considering these situations, as shown in Figure 6.

BPTL aims to jointly optimize and recursively iterate learning between logical and perceptual models by returning logical labels through logical modules. It resolves conflict problems arising from symbol transformation in logical reasoning until *z* can be corrected to $z^{*}$ such that $\Delta_{\phi }\left[\left\{z^{*}\right\}\right]⊧y$.

We further harmonized the definitions of terms and formulas, as they have the same recursive format. For each step of the recursion, we denoted the set of inputs (atomic terms/formulas) as $S^{\left(i\right)}$, the set of operations/relationships/connectors as $\Psi_{s}$, and the set of outputs can be denoted as

(5) $$S^{\left(o\right)}=\left\{\psi \left(s_{1},s_{2},\ldots s_{r_{f}}\right)|\psi \in \Psi_{s},s_{i}\in S^{\left(i\right)}\right\}$$

By denoting the above process of finding a valid $z^{*}$ from *z* as $Q(z|z^{*})$, we define an acceptance probability as follows:

(6) $$\alpha \left(z,z^{*}\right)=\frac{\pi \left(z^{*}\right)Q\left(z|z^{*}\right)}{\pi \left(z^{*}\right)Q\left(z|z^{*}\right)+\pi \left(z\right)Q\left(z^{*}|z\right)}$$

In practice, if $\Delta_{\phi }\left[z\right]\neq y^{*}$ then $Q(z|z^{*})$ may be 0. The acceptance probability is computed by adding a parameter δ to $Q(z|z^{*})$, and then we can obtained the following three conditions:

(1) $\pi \left(z\right)=0\&\pi \left(z^{*}\right)>0\Rightarrow \alpha \left(z,z^{*}\right)=1$ (always accept).
(2) $\pi \left(z\right)=0\&\pi \left(z^{*}\right)=0\Rightarrow \alpha \left(z,z^{*}\right)=0$ (always reject).
(3) $\pi \left(z\right)>0\&\pi \left(z^{*}\right)>0\Rightarrow \alpha \left(z,z^{*}\right)\in \left(0,1\right)$ (conditional).

In Figure 6, both BPCs (Backpropagate Constraints) and BPTLs (Backpropagate Through Logics) are used to solve conflict problems in logical reasoning, but they differ in their specific roles.

BPCs ensure that the transformation of logical symbols satisfies given logical relationships and constraints by applying constraints during backpropagation. They play a crucial role in preventing the generation of inconsistent or invalid reasoning results, thus enhancing the accuracy and reliability of logical reasoning. BPTLs match target labels by recursively backpropagating and adjusting symbols to ensure that the final symbol representation satisfies the predictions of the logical model. They help to resolve conflicts and inconsistencies due to symbol transformations in logical reasoning, thereby enhancing the learning and reasoning capabilities of the overall model. For instance, if two different paths attempt to modify the same symbol to inconsistent values, the algorithm needs to resolve such conflicts rationally. At each step of the symbol transformation from *z* to $z^{*}$, the algorithm computes an acceptance probability to ensure that the final symbol representation $z^{*}$ probabilistically matches the predictions of the logic model. The structure of the deep logic module is utilized to recursively backtrack from output to input, thereby adjusting symbols layer by layer to match the target label. The most appropriate symbol transformation path is selected through random sampling and logic model-based evaluation, with acceptance probabilities ensuring the optimization of the selected path.

Through Figure 6, the transformation from sensory data (e.g., images of electrical equipment) to logical causal reasoning of faults can be efficiently integrated and optimized to improve the performance and generalization of the model in complex logic tasks.

#### 3.2.3. Attention Mechanism Driven Historical Event Embedding Module

We employed an attention-based gated loop module to encode the information sequential patterns of historical events. The primary objective of this module is to model the pattern of historical events between pairs of entities, thereby encoding temporal information and generating temporal embeddings corresponding to entities and relations. For events (s,r,o,t) and their inverse events $\left(o,r^{-1},s,t\right)$, the underlying temporal features and patterns embedded in historical events can reveal historical trends and patterns. To adequately capture the temporal patterns of these historical events, the model needs to fully consider the temporal sequence of events. To enhance the effectiveness of capturing information related to equipment failures, we introduced a historical event attention mechanism. This mechanism enables the module to dynamically select and linearly combine the relationships between different historical events. The specific formulas are described below:

(7) $$\begin{array}{c} e_{\tau }=v_{e}^{T}\tanh\left(W_{e}r_{t}+U_{e}r_{\tau }\right) \end{array}$$

(8) $$\begin{array}{c} a_{\tau }=\mathrm{softmax}\left(e_{\tau }\right)\frac{\exp\left(e_{\tau }\right)}{\sum_{\tau =1}^{t}\exp\left(e_{\tau }\right)} \end{array}$$

(9) $$\begin{array}{c} r_{t}=\sum_{\tau =1}^{t}a_{\tau }r_{\tau } \end{array}$$

where $v_{e}^{T}$, $W_{e}$, and $U_{e}$ are parameters, and $a_{\tau }$ is used to determine which part of the historical event to focus on in the prediction. The relational embedding $r_{t}$ forms the embedding matrix $R_{t}$ of the relation at *t*.

#### 3.2.4. Model Prediction

The main method of applying knowledge reasoning in entity reasoning and relationship reasoning tasks is to calculate the probability of the occurrence of entities or relationships. In this paper, we used ConvTransE [39] to calculate the probability of entities and relationships as shown in Equations (10) and (11):

(10) $$\begin{array}{cc} \; & p\left(o|H_{t-1},R_{t-1},s,r\right)=\mathrm{sigmoid}\left(H_{t-1}\mathrm{ConvTrans}E\left(s_{t-1},r_{t-1}\right)\right) \end{array}$$

(11) $$\begin{array}{cc} \; & p\left(r|H_{t-1},R_{t-1},s,o\right)=\mathrm{sigmoid}\left(R_{t-1}\mathrm{ConvTrans}E\left(s_{t-1},o_{t-1}\right)\right) \end{array}$$

where $s_{t-1}$, $r_{t-1}$, and $o_{t-1}$ denote the vector form of the event subject *s*, the relation *r*, and the event object *o* at timestamp t−1 in $H_{t-1}$ and $R_{t-1}$, respectively.

In the process of electrical fault detection, the first step is to collect detailed information related to the fault. This information is then deeply parsed to transform it into a standard quaternion form that can be easily represented using a temporal knowledge graph. Next, the query function of the graph database Neo4j is utilized to search the knowledge graph for historical records that are similar to the current fault. If a similar case is found, the data encoder and decoder will be used to quickly obtain the prediction information related to the current fault. However, if a similar case is not detected, the fault is subjected to analysis by experts. In such cases, the knowledge graph is queried to yield exhaustive analysis information, which in turn furnishes robust support for fault resolution processes.

## 4. Results

### 4.1. Verification of State Detection Methods for Power Equipment

**Figure 7:**
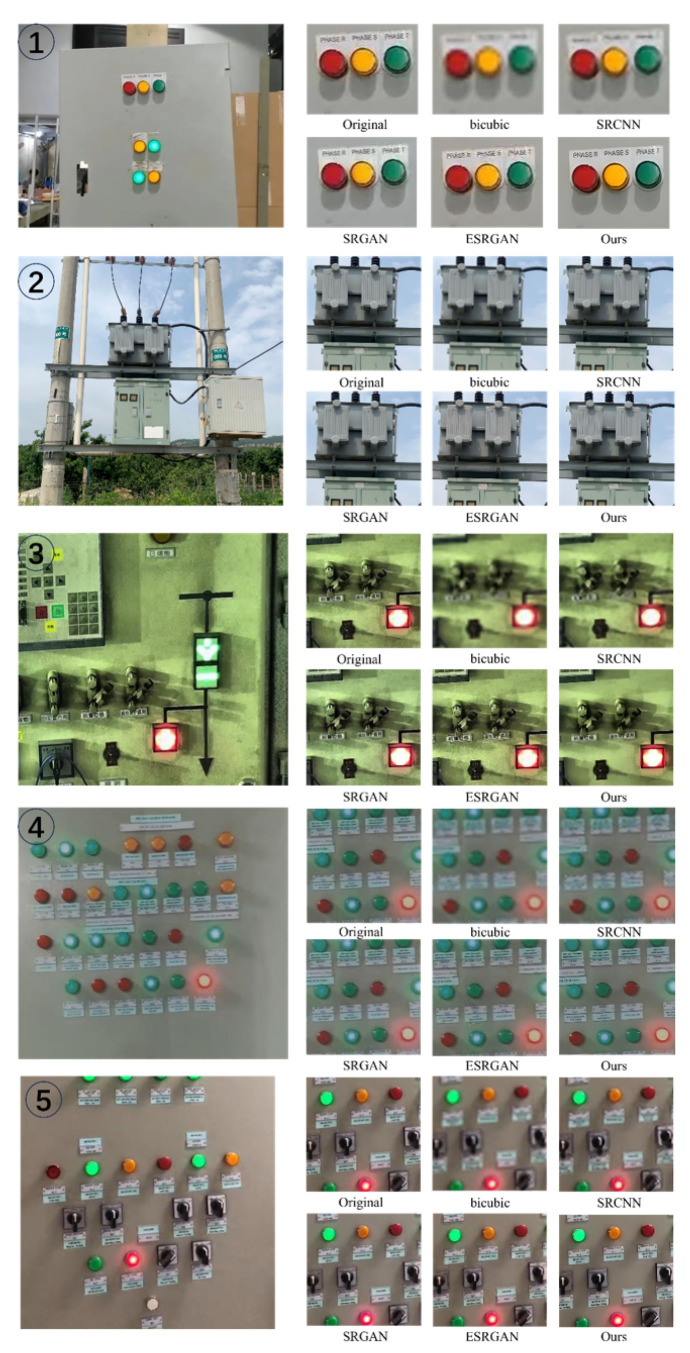
Comparison of super-resolution reconstruction effects using different methods on five different electrical devices.

To further enhance the resolution of electrical equipment images and augment the detailed information within them, we employed an improved super-resolution reconstruction method. This method is designed to refine electrical equipment images through advanced algorithms and techniques, thus resulting in a significant improvement in the clarity and resolution of the images. In Figure 7, we compare our method with other super-resolution reconstruction methods, which include SRCNN, SRGAN, and ESRGAN [40]. Moreover, to facilitate a quantitative comparison between different methods, we constructed a dataset containing 15,000 noisy image samples, thereby focusing on substation equipment such as power panels, control boxes, and transformers. During the dataset construction, the blurred images were caused by camera shake and poor lighting conditions. All images were captured using an 8-megapixel camera under consistent settings, with a shooting distance of 3 to 5 m and automatic adjustment of the camera’s focus. For evaluation metrics, we selected the peak signal-to-noise ratio (PSNR), structural similarity (SSIM), and natural image quality evaluator (NIQE) [41], and the comparison results are shown in Table 1.

Through the comparison in Figure 7, it is evident that our method outperformed the previous approach in eliminating blurring and artifacts surrounding the power control panel buttons. Additionally, it excelled in recovering the texture and details of the characters, which significantly improved visual clarity. Further observing the values of different evaluation metrics in Table 2, we can find that although the reconstructed images using the ESRGAN method had relatively higher PSNR and SSIM values, our method had little difference in the PSNR and SSIM values compared to the ESRGAN method and performed better in the NIQE values, which means that our reconstructed images are of higher quality.

### 4.2. Failure Rate Identification of Electrical Equipment

**Figure 8:**
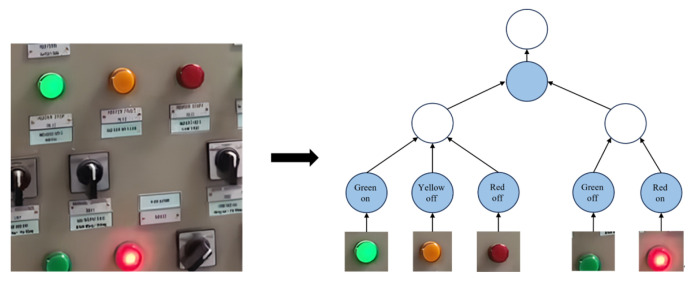
Schematic diagram for identifying the abnormal state of power switchgear equipment.

Taking the switchgear in the power system as an example, the operation of the system can be inferred by monitoring the status of the switchgear buttons. In the switchgear cabinet illustrated in Figure 8, two modules are included: the green indicator of the first module lights up continuously, while the red indicator of the second module also lights up continuously. Through logical reasoning, it can be concluded that the system is in an abnormal state. This state indicates that there may be a fault or abnormal condition that requires further inspection and treatment to ensure the safe and stable operation of the power system.

**Figure 9:**
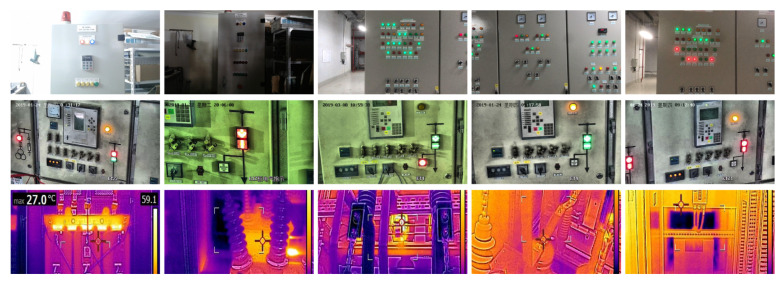
Image samples for the electrical equipment.

We constructed six datasets (system undervoltage, system voltage overload, system current overload, panel or line overheating, key phase abnormality, no influence abnormality, and panel display abnormality) to provide diverse fault scenarios. Each dataset contains 2000 data samples, and the data samples contain information on the device types, device number, button phase, panel light, surface temperature, and the desired system state. Part slices of the data samples are shown in Figure 9. Then, we compared the method we proposed with other commonly used methods to evaluate their performance in the task of electrical equipment fault rate recognition. The results are shown in Table 3. These comparison tests aim to validate the effectiveness and superiority of our method and provide strong support for further research.

By analyzing the data in the table, it becomes evident that our proposed method performed more superiorly in terms of fault recognition accuracy compared to the other three commonly used methods on six electrical equipment anomaly datasets. For abnormal state diagnosis on timing information such as voltage or current, the performance of our method was very close to that of other SOTA time series forecast methods. In predicting sparsely varying device voltages, our method showed some superiority. However, in the case of more densely varying device currents, the performance was slightly worse than that of the timing prediction networks such as LSTM and GRU. This is because our method is essentially based on a probabilistic model, which is less sensitive to long time variations. For more complex and trivial system states, such as specific button misalignment and panel display anomalies, our algorithm greatly outperformed other SOTA methods, mainly because our algorithm has the ability of logical deduction, which can map the system state of power equipment to specific atomic nodes through the C-AOG graph, and thanks to the strengthening of this logical correlation between the state of the buttons or the panel state and the state of the system, we can predict the state of the power equipment by using the C-AOG graph. Our diagnostic method is more sensitive to the misalignment of buttons and display anomalies of the panels. It is worth noting that, in addition to the high recognition rate, our method also has high interpretability that provides interpretation and understanding of the fault recognition results. This suggests that our method not only has higher applicability and effectiveness in electrical equipment fault identification tasks but also provides users with more intuitive and credible diagnostic results.

## 5. Conclusions

In this article, we have dedicated its work to solving the problems of low image quality and the lack of logical deduction ability in deep learning-based electrical equipment fault state recognition methods. We proposed an electrical equipment image quality enhancement method that effectively enhances the quality of electrical equipment images by integrating image light balancing with super-resolution reconstruction techniques. Additionally, we introduced an interpretable electrical equipment fault logic reasoning method by combining the C-AOG-based system state change derivation approach with BPTL and integrating a history embedding module driven by an attention mechanism. The experimental results demonstrate the superiority of our proposed image quality enhancement method in eliminating blurring and artifacts around power control panel buttons compared to alternative methods. Moreover, our method exhibited enhanced capability in recovering texture and character details. On the six abnormal datasets of power equipment we constructed, our proposed method outperformed the other three commonly used methods in terms of the fault recognition rate and interpretability.

The research in this paper also has some shortcomings. In our study, we mainly performed fault diagnosis by capturing external features such as the appearance of the device, panel, buttons, temperature characteristics, etc., and we did not consider the internal features of some devices, which makes it difficult for our method to be applied to some special scenarios. In addition, as a class of probabilistic inference models, the fault inference model built based on C-AOG suffered from the problem of low knowledge granularity. Therefore, when dealing with continuous dense data, the diagnostic performance of our method was slightly weaker than some SOTA time series analysis algorithms.

## Figures and Tables

**Table 1:** The abbreviations list.

Abbreviation	Meaning
GAN	Generative Adversarial Network
SVM	Support Vector Machine
CNN	Convolutional Neural Network
RNN	Recurrent Neural Network
LSTM	Long Short-Term Memory Network
SCM	Structural Causal Model
HR	High-Resolution
RRDB	Residual-in-Residual Dense Block
C-AOG	Causal And-Or Graph Model
BPCs	Backpropagate Constraints
BPTLs	Backpropagate Through Logic

**Table 2:** Quantitative comparison of our method with other methods on different evaluation indicators.

Order	Index	Bicubic	SRCNN	SRGAN	ESRGAN	Ours
➀	PSNR	35.84	35.83	31.94	**37.78**	36.74
	SSIM	0.9019	0.91	0.9425	**0.9465**	0.9420
	NIQE	4.6947	4.1238	4.2027	3.5957	**3.4186**
➁	PSNR	32.36	32.42	30.91	**33.21**	32.30
	SSIM	0.7277	0.7560	0.7743	0.8414	**0.8430**
	NIQE	4.1125	3.4626	3.0537	**2.6053**	2.6497
➂	PSNR	30.73	30.75	30.45	**31.52**	31.24
	SSIM	0.6020	0.6215	**0.6949**	0.6879	0.6825
	NIQE	4.5287	4.2239	5.0030	3.2444	**2.2891**
➃	PSNR	35.36	35.71	33.01	34.33	**36.55**
	SSIM	0.8896	0.9059	0.9908	**0.9964**	0.9579
	NIQE	4.2233	3.9362	4.1759	3.3094	**3.2087**
➄	PSNR	35.40	35.35	31.38	**36.35**	35.27
	SSIM	0.8854	0.8955	0.9104	**0.9152**	0.8894
	NIQE	4.7789	4.1981	3.8177	3.3387	**3.2100**

**Table 3:** Electrical equipment failure recognition accuracy comparison.

Dataset	RF [42]	LSTM [18]	GRU [43]	Ours(C-AOG+SR)
System Undervoltage	79.4%	80.2%	82.5%	**83.1%**
System Voltage Overload	80.7%	84.4%	86.3%	**87.5%**
System Current Overload	81.2%	82.8%	**83.4%**	82.6%
Panel or Line Overheating	91.3%	93.6%	95.7%	**96.4%**
Button Phase Abnormality	71.2%	75.9%	76.8%	**83.2%**
Panel Display Abnormality	76.9%	78.5%	79.3%	**82.6%**

## Data Availability

Data are contained within the article.
